# Changes in the methodology of medical teaching due to the COVID-19 pandemic

**DOI:** 10.31744/einstein_journal/2023AO0101

**Published:** 2023-07-25

**Authors:** Alexandre Pedro Nicolini, Fausto Santana Celestino, Carlos Eduardo da Silveira Franciozi, Carlos Vicente Andreoli, Nacime Salomão Barbachan Mansur

**Affiliations:** 1 Universidade Federal de São Paulo São Paulo SP Brazil Universidade Federal de São Paulo, São Paulo, SP, Brazil.

**Keywords:** Learning, Teaching, School teachers, Students, Education, distance, Education, medical, COVID-19, Pandemics, Motivation, Surveys and questionnaires

## Abstract

**Objective:**

To evaluate the perceptions of students and teachers regarding remote teaching modality in comparison with the traditional face-to-face method.

**Methods:**

In this observational, retrospective, comparative, single-center study, questionnaires containing three major assessment domains were sent to two groups: university professors and undergraduate and graduate students. The first domain collected demographic and general data on the platforms used. The second and third domains contained questions that compared the perception of the quality of information offered by the two systems.

**Results:**

Between May and September 2020, 162 students and 71 teachers participated in the study. A greater proportion of students demonstrated previous contact with the online method, while professors had presented a greater number of courses. Most participants reported that their expectations regarding the remote teaching method were met (students, 80.3%; teachers, 94.4%). A significant number of students (83.3%) and teachers (88.7%) rated the classes as easier to attend and manage. Despite difficulties, such as concentration retention, most of the participants agree (at least partially) that the format should be maintained.

**Conclusion:**

The remote teaching methodology, although still incipient in Brazil, has become a reality in light of current health restrictions. Our study demonstrated a high level of overall satisfaction and a high sense of learning from both students and faculty. However, new challenges associated with this system have been identified, such as retention of attention and interference from the external environment. Longitudinal comparative studies that incorporate various aspects of medical education in all cycles are necessary to corroborate the findings of this study.

**Design:**

Retrospective comparative study, level III evidence.

**Figure:**
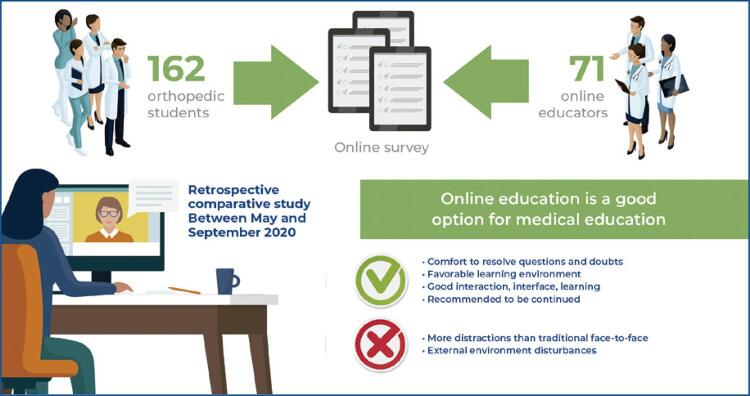

## INTRODUCTION

Until recently, the vast majority of academic courses were conducted in person, using a methodology centered on the teacher, who is responsible for transmitting all of his knowledge to students who passively attend class.^( 1 - 4 )^ Previous studies have shown that programs that focus on teachers tend not to promote lasting learning.^( 1 , 2 , 5 )^

There is a current proposal to redirect medical education to a student-centered methodology rather than solely relying on the teacher. This approach encourages students to participate more actively in the learning process, promoting greater engagement and, consequently, more lasting learning.^( 1 - 4 , 6 - 8 )^ To this end, several methodologies have been employed that have the common characteristic of including direct student participation, such as teaching based on clinical case studies, group discussions, encouraging critical thinking, availability of study material before class, project development, and competition.^( 1 , 9 , 10 )^

Online teaching is part of an even larger medical practice called digital education. Technology provides medical education with the possibility of remote teaching, simulations (including surgeries), and the use of applications and/or new technological equipment.^( 3 , 11 - 13 )^ The investments in several educational institutions for the promotion and development of digital education are increasing. Despite this exponential increase in spending and use of digital education, there is a lack of evidence supporting its use in the training of health professionals.^( 11 , 12 )^

This methodology offers greater flexibility, usually at a lower cost to students, and has the advantage of providing material to a greater number of people. Even from a distance, the public can attend courses, classes, and congresses simply by accessing the Internet via cell phones or computers.^( 2 , 5 , 13 - 19 )^ Other advantages of this online methodology include the possibility of recording the classes, making access to content practically infinite, and the ability for guests from other institutions to teach a class without being constrained by distance.^( 2 )^

Online teaching, which has been used for some time, is gaining increasing attention in universities worldwide.^( 13 )^ In 2015, 29.7% of US higher education students attended at least one course online, which was 3.9% higher than in the previous year.^( 15 )^

A 2013 study showed that 33.5% of higher education students had taken at least one online course, totaling 7.1 million students.^( 20 )^ In Brazil, there are no statistics on this percentage; however, participation is believed to be lower.

Medical education using digital platforms has become extremely relevant in the face of the COVID-19 pandemic in 2020. Teaching, which for the most part was carried out in person, was abruptly replaced by digital teaching, giving students and teachers first contact with this method of teaching.

## OBJECTIVE

This study aimed to investigate the online teaching methodologies used by students and teachers and compare them to conventional classroom teaching methodologies. Additionally, we aimed to evaluate the benefits and criticisms of the online teaching method to provide tools and information for its improvement.

## METHODS

### Design

This retrospective comparative study was conducted between May and September 2020. After receiving approval from the Institutional Ethics Committee of *Universidade Federal de São Paulo* (UNIFESP) and *Plataforma Brasil* (# 4560090420), the authors sent questionnaires to UNIFESP students and professors. The questionnaires contained an informed consent form (ICF) that needed to be completed.

The study sample included professors with contracts in the medical course and students who were regularly enrolled in undergraduate medical courses, medical residency, and *lato-sensu* postgraduate courses at the same university. Students from other courses, professors not working in medical courses, and individuals who did not complete the survey were excluded.

### Measurements

The two questionnaires were designed to target different populations. Both surveys comprised questions about general epidemiological data and direct multiple-choice questions aimed at inquiring about the various facets and characteristics of distance learning that had been or were being carried out. These questions asked about the classroom environment, platforms used, the possibility of interaction and doubts, and general experience.

### Statistical analysis

Participants’ quantitative variables were described using mean and standard deviation and compared between groups using the Student’s *t* -test. Other qualitative characteristics were described according to the groups using absolute and relative frequencies. The association between groups was assessed using the χ^2^ test or exact tests (Fisher’s exact test or the likelihood ratio test). For characteristics whose responses were formulated using the Likert scale (five graded categories), the groups were compared using the Mann-Whitney test. Data analyses were performed using SPSS for Windows version 20.0 (SPSS Inc., Chicago, Illinois, USA) and tabulated using Microsoft Excel 2003. The tests were performed at a significance level of 5%.

## RESULTS

A total of 162 students and 71 teachers were included in this study from May to September 2020. The demographic characteristics of the participants are presented in table 1 . Not all participants answered all the questions.

**Table 1:** Description of personal characteristics and courses taken/taught

Variable	Groups		Total (n=233)	p value

	Students (n=162)	Teachers (n=71)		
Age (years), (Average/standard deviation)	30.8	478.6	35.711.8	<0.001
Sex, n (%)				<0.001
Female	48 (29.6)	6 (8.6)	54 (23.3)	
Male	114 (70.4)	64 (91.4)	178 (76.7)	
Courses, n (%)				<0.001
Only one	162 (100)	23 (32.9)	185 (79.7)	
More than one	0 (0)	47 (67.1)	47 (20.3)	
Local, n (%)				0.084
Home	105 (64.8)	38 (54.3)	143 (61.6)	
Work	5 (3.1)	3 (4.3)	8 (3.4)	
Both	51 (31.5)	25 (35.7)	76 (32.8)	
Another place	1 (0.6)	4 (5.7)	5 (2.2)	
Preview experience, n (%)				0.018
No	84 (51.9)	48 (68.6)	132 (56.9)	
Yes	78 (48.1)	22 (31.4)	100 (43.1)	
Number of platforms used, n (%)				0.411
1	24 (15)	10 (14.3)	34 (14.8)	
2	44 (27.5)	28 (40)	72 (31.3)	
3	64 (40)	19 (27.1)	83 (36.1)	
4	26 (16.3)	10 (14.3)	36 (15.7)	
5	2 (1.3)	3 (4.3)	5 (2.2)	
Which platform do you most like, n (%)				<0.001
Google Hangouts^®^	1 (0.6)	6 (8.6)	7 (3)	
Google Meet^®^	86 (53.8)	17 (24.3)	103 (44.8)	
GoToWebinar^®^	2 (1.3)	2 (2.9)	4 (1.7)	
Zoom^®^	63 (39.4)	41 (58.6)	104 (45.2)	
Slack^®^	0 (0)	1 (1.4)	1 (0.4)	
Another one	8 (5)	3 (4.3)	11 (4.8)	
Device, n (%)				<0.001
Computer / Notebook	24 (15.1)	42 (60)	66 (28.8)	
Smartphone / Tablet	8 (5)	0 (0)	8 (3.5)	
Both	127 (79.9)	28 (40)	155 (67.7)	

The data in this table are included in the Student’s *versus* Teachers’ Questionnaire Part 1.

Students commonly took only one course, while more than 50% of the teachers taught more than one virtual course (p<0.001). Students had significantly more prior participation in remote courses than professors (p=0.018). Among teachers, the preferred platform was Zoom^®^, while for students, it was Google Meet^®^ (p<0.001). The use of portable devices (smartphones and tablets) was significantly higher among students (p<0.001).

The analysis was then conducted on expectations regarding remote teaching compared to face-to-face teaching ( Table 2 ). Teachers reported feeling more comfortable with healing than students (p=0.007).

**Table 2:** Description of the characteristics of remote teaching expectations according to groups and results of comparative tests

Variable	Groups		Total (n=233)	p value

	Students (n=162)	Teachers (n=71)		
Previous expectation was met				0.202
Strongly agree	44 (27.2)	18 (25.4)	62 (26.6)	
Somewhat agree	86 (53.1)	49 (69)	135 (57.9)	
Neither agree nor disagree	12 (7.4)	3 (4.2)	15 (6.4)	
Somewhat disagree	13 (8)	1 (1.4)	14 (6)	
Strongly disagree	7 (4.3)	0 (0)	7 (3)	
Comfortable to clear doubts				0.007
Strongly agree	61 (37.7)	35 (49.3)	96 (41.2)	
Somewhat agree	60 (37)	31 (43.7)	91 (39.1)	
Neither agree nor disagree	6 (3.7)	3 (4.2)	9 (3.9)	
Somewhat disagree	24 (14.8)	1 (1.4)	25 (10.7)	
Strongly disagree	11 (6.8)	1 (1.4)	12 (5.2)	
Classroom environment conducive				0.823
Strongly agree	56 (34.6)	16 (22.5)	72 (30.9)	
Somewhat agree	62 (38.3)	46 (64.8)	108 (46.4)	
Neither agree nor disagree	18 (11.1)	6 (8.5)	24 (10.3)	
Somewhat disagree	13 (8)	2 (2.8)	15 (6.4)	
Strongly disagree	13 (8)	1 (1.4)	14 (6)	
External environment makes it difficult				0.798
Strongly agree	44 (27.2)	15 (21.1)	59 (25.3)	
Somewhat agree	64 (39.5)	39 (54.9)	103 (44.2)	
Neither agree nor disagree	15 (9.3)	3 (4.2)	18 (7.7)	
Somewhat disagree	28 (17.3)	10 (14.1)	38 (16.3)	
Strongly disagree	11 (6.8)	4 (5.6)	15 (6.4)	
Good interaction: students *versus* teacher				0.395
Strongly agree	40 (24.7)	7 (9.9)	47 (20.2)	
Somewhat agree	74 (45.7)	43 (60.6)	117 (50.2)	
Neither agree nor disagree	6 (3.7)	11 (15.5)	17 (7.3)	
Somewhat disagree	29 (17.9)	8 (11.3)	37 (15.9)	
Strongly disagree	13 (8)	2 (2.8)	15 (6.4)	
Good interaction between students				0.357
Strongly agree	34 (21)	7 (9.9)	41 (17.6)	
Somewhat agree	39 (24.1)	26 (36.6)	65 (27.9)	
Neither agree nor disagree	18 (11.1)	20 (28.2)	38 (16.3)	
Somewhat disagree	36 (22.2)	12 (16.9)	48 (20.6)	
Strongly disagree	35 (21.6)	6 (8.5)	41 (17.6)	
Class method allows for more distractions				0.819
Strongly agree	57 (35.2)	23 (32.4)	80 (34.3)	
Somewhat agree	64 (39.5)	33 (46.5)	97 (41.6)	
Neither agree nor disagree	15 (9.3)	7 (9.9)	22 (9.4)	
Somewhat disagree	14 (8.6)	7 (9.9)	21 (9)	
Strongly disagree	12 (7.4)	1 (1.4)	13 (5.6)	
Learning *versus* Apprenticeship				0.624
Strongly agree	39 (24.1)	9 (12.7)	48 (20.6)	
Somewhat agree	80 (49.4)	45 (63.4)	125 (53.6)	
Neither agree nor disagree	19 (11.7)	15 (21.1)	34 (14.6)	
Somewhat disagree	17 (10.5)	1 (1.4)	18 (7.7)	
Strongly disagree	7 (4.3)	1 (1.4)	8 (3.4)	
Adequate resources				0.920
Strongly agree	60 (37)	17 (23.9)	77 (33)	
Somewhat agree	66 (40.7)	49 (69)	115 (49.4)	
Neither agree nor disagree	15 (9.3)	1 (1.4)	16 (6.9)	
Somewhat disagree	15 (9.3)	4 (5.6)	19 (8.2)	
Strongly disagree	6 (3.7)	0 (0)	6 (2.6)	
Recommendation to keep				0.287
Strongly agree	42 (25.9)	15 (21.1)	57 (24.5)	
Somewhat agree	60 (37)	38 (53.5)	98 (42.1)	
Neither agree nor disagree	21 (13)	10 (14.1)	31 (13.3)	
Somewhat disagree	23 (14.2)	7 (9.9)	30 (12.9)	
Strongly disagree	16 (9.9)	1 (1.4)	17 (7.3)	

The data were included in the Student *versus* Teacher Questionnaire Part 2, an online class evaluation.

Regarding the positive factors, both groups mostly agreed that the method met expectations, provided a comfortable way to resolve doubts (74.7% students *versus* 93% teachers), created a favorable learning environment, and allowed for good interaction between students and teachers. Additionally, both groups reported that the method facilitated good use and learning, considered the appropriate resources, and that they would recommend and maintain in this format. Regarding the negative factors of remote teaching, both groups mostly agreed that the external environment made it difficult and that the method allowed for more distractions.

The following were considered regarding the features of interest and environment ( Table 3 ). Students mostly chose that the external environment is “much worse” compared to teachers, and this difference was statistically significant (p=0.045). Students also chose that maintaining attention is “much worse,” unlike teachers (p<0.001).

**Table 3:** Description of the characteristics of interest and environment of remote teaching according to groups and results of comparative tests

Variable	Groups		Total (n=233)	p value

	Students (n=162)	Teachers (n=71)		
Causes an interest in the topic				0.246
Much worse	14 (8.6)	0 (0)	14 (6)	
Worse	40 (24.7)	20 (28.2)	60 (25.8)	
Equal	76 (46.9)	33 (46.5)	109 (46.8)	
Better	18 (11.1)	15 (21.1)	33 (14.2)	
Much better	14 (8.6)	3 (4.2)	17 (7.3)	
Interaction teacher *versus* students				0.347
Much worse	16 (9.9)	3 (4.2)	19 (8.2)	
Worse	76 (46.9)	47 (66.2)	123 (52.8)	
Equal	49 (30.2)	11 (15.5)	60 (25.8)	
Better	14 (8.6)	8 (11.3)	22 (9.4)	
Much better	7 (4.3)	2 (2.8)	9 (3.9)	
Interaction between students				0.356
Much worse	31 (19.1)	3 (4.2)	34 (14.6)	
Worse	78 (48.1)	49 (69)	127 (54.5)	
Equal	39 (24.1)	10 (14.1)	49 (21)	
Better	6 (3.7)	5 (7)	11 (4.7)	
Much better	8 (4.9)	4 (5.6)	12 (5.2)	
Possibility to resolve doubts				0.403
Much worse	11 (6.8)	0 (0)	11 (4.7)	
Worse	33 (20.4)	30 (42.3)	63 (27)	
Equal	89 (54.9)	25 (35.2)	114 (48.9)	
Better	20 (12.3)	12 (16.9)	32 (13.7)	
Much better	9 (5.6)	4 (5.6)	13 (5.6)	
Class environment				0.706
Much worse	13 (8)	0 (0)	13 (5.6)	
Worse	47 (29)	26 (36.6)	73 (31.3)	
Equal	44 (27.2)	22 (31)	66 (28.3)	
Better	45 (27.8)	14 (19.7)	59 (25.3)	
Much better	13 (8)	9 (12.7)	22 (9.4)	
External environment				0.045
Much worse	22 (13.6)	1 (1.4)	23 (9.9)	
Worse	69 (42.6)	34 (47.9)	103 (44.2)	
Equal	43 (26.5)	19 (26.8)	62 (26.6)	
Better	22 (13.6)	9 (12.7)	31 (13.3)	
Much better	6 (3.7)	8 (11.3)	14 (6)	
Ease of attending / giving class				0.801
Much worse	8 (4.9)	0 (0)	8 (3.4)	
Worse	9 (5.6)	1 (1.4)	10 (4.3)	
Equal	10 (6.2)	7 (9.9)	17 (7.3)	
Better	47 (29)	29 (40.8)	76 (32.6)	
Much better	88 (54.3)	34 (47.9)	122 (52.4)	
Keeping attention / ease of preparing class				<0.001
Much worse	22 (13.6)	0 (0)	22 (9.4)	
Worse	55 (34)	2 (2.8)	57 (24.5)	
Equal	55 (34)	53 (74.6)	108 (46.4)	
Better	15 (9.3)	6 (8.5)	21 (9)	
Much better	15 (9.3)	10 (14.1)	25 (10.7)	
Sense of learning				0.854
Much worse	13 (8)	0 (0)	13 (5.6)	
Worse	33 (20.4)	26 (36.6)	59 (25.3)	
Equal	91 (56.2)	31 (43.7)	122 (52.4)	
Better	16 (9.9)	11 (15.5)	27 (11.6)	
Much better	9 (5.6)	3 (4.2)	12 (5.2)	
General assessment				0.742
Much worse	14 (8.6)	0 (0)	14 (6)	
Worse	48 (29.6)	27 (38)	75 (32.2)	
Equal	53 (32.7)	24 (33.8)	77 (33)	
Better	38 (23.5)	17 (23.9)	55 (23.6)	
Much better	9 (5.6)	3 (4.2)	12 (5.2)	

The data were included in the Student *versus* Teacher Questionnaire Part 3, a comparison with face-to-face classes.

Regarding the positive factors, both groups reported that the classes were easier to attend and administer in a virtual format. Regarding negative factors, both groups agreed that the interaction between teachers and students was worse or much worse and that the external environment was worse or much worse during the classes. Concerning the feeling of learning, most students and teachers considered the virtual format similar to the face-to-face format. However, a reasonable portion considered this feeling to be worse or much worse.

## DISCUSSION

In the face of the pandemic caused by the new coronavirus, many in-person academic activities in medical courses have now been carried out virtually. Despite this sudden and necessary change, little has been reported on how teaching is carried out using this method and the perceptions of the teacher-student binomial in this reality. Our study showed that, according to the participants, many qualities of face-to-face teaching were maintained in remote learning. Some factors were rated better, and others worse when virtual rooms were used.

Although remote teaching is common in other countries, in Brazil, there is no expressive participation, as demonstrated by our results which show that 51.9% of students and 68.6% of teachers had never participated in online classes before the pandemic. This highlights the decisive role that the COVID-19 pandemic has played in bringing about methodological changes in teaching. In general, our study showed that the experience at our university and in our population of students and professors was considered positive, with expectations met regarding a comfortable environment that was conducive to learning, with good use, and an interactive environment. Despite these difficulties, most online methods have been recommended and maintained. Furthermore, our findings are consistent with previous studies.^( 3 , 13 - 15 , 19 , 21 )^

Some aspects of the online method were identified as unfavorable, such as the external environment that can interfere, the greater probability of distraction, and a certain decrease in the interaction between teachers and students and between students themselves. Although not in the majority, a considerable proportion of the groups considered the method to be worse and had a worse sense of learning.

Comparing our findings to those in the literature owing to several factors, including the considerable variability of the populations studied, with important differences in the social and economic context, and variation in the criteria considered most important, which are given greater prominence when comparing methods. Thus, some results may appear discordant; however, there is a general tendency to consider the online method to be similar or better.

Monier et al.^( 14 )^ analyzed students’ perceptions of a particular course that was conducted remotely. They obtained a sample of 319 students, 65% of whom rated the course as excellent, 34% as good, and only 1% as poor. The authors suggested that tutors play a key role and must provide adequate support for the good performance and motivation of students in remote courses. Harwood et al.^( 15 )^ cited several advantages of online teaching, such as greater accessibility, greater flexibility, and lower cost. In the same article, they discussed two important studies that compared face-to-face and remote methodologies, one of them a systematic review conducted by Cook et al.^( 22 )^ In this review authors analyzed 76 studies and found similar efficacies between the two methodologies. The other study was conducted by Reis et al,^( 21 )^ with 40 physicians comparing the same methodologies and found that 86% of students found the online method superior, specifically in promoting student-teacher interaction, increasing interest in the subject, and promoting motivation. In 2008, Cook et al.^( 22 )^ conducted a new meta-analysis of 2001 studies and reported results similar to those of their previous work, with no significant difference between the methods.

Pei et al.^( 13 )^ performed a meta-analysis comparing face-to-face and online teaching using only medical students, excluding graduate students, and suggested that they eliminated the maturity factor as a motivational bias favorable to remote teaching. They analyzed 16 studies and reported that 7 studies showed no differences were found between the two methodologies, and in 9, they found an advantage for online learning. However, the authors criticized the evaluations conducted, and in most cases, they did not evaluate long-term learning. They also suggested that blended learning (face-to-face and online), combining the qualities of both, could often be the most interesting way of teaching.

El Sayed et al.^( 23 )^ conducted a comparative study (with objective and subjective data collected) to evaluate a group that had classes exclusively in person and a group that had classes exclusively online, both using the same pedagogical material. They concluded that satisfaction with the course, teacher and student assessment methods, and test scores (discursive and oral) were similar between the groups. However, the group that took online classes had a significantly higher average grade in course completion (p<0.001). This difference was attributed to the fact that the method saves students time and improves their engagement.^( 23 )^

Regarding teachers, we can suggest tools that help facilitate the learning process during this difficult and abrupt transition in methodology.^( 1 , 20 )^ Based on the population we studied, the biggest challenges are related to maintaining the attention of the student group and capturing their interest, preparing the external environment in a way that minimizes distractions, and making the class more interactive.

Jiang et al. suggested the following as good practices for online teaching: 1) promoting small and more interactive groups with pre- and post-class discussions, as active peer participation and teacher feedback increase learning ability; 2) seeking a more sociable environment, a more positive atmosphere, and making the group more cohesive; 3) using learning quality assessment tools, for example, evaluating students with clinical cases, rather than grades and rankings; and 4) incorporating clinical simulation in the education process-problem-based learning.^( 24 )^ Lewis et al.^( 25 )^ qualitatively evaluated the characteristics considered important in online courses for physicians. They noted that most teachers and students were unfamiliar with digital teaching platforms, and it was not enough to transmit the conventional class prepared for the face-to-face format online for effective learning. Moreover, they emphasized the need to prepare classes in a more interactive way that is appropriate for the new scenario. Teachers experimented with new ways of engaging students to encourage interaction in the online environment, such as the problem-based learning method, in which students gain knowledge and skills by extensively investigating to work individually and in groups (to solve a problem). Additionally, the facilitative teaching style, in which the teacher guides, instigates, motivates, and serves as a catalyst rather than the source of learning. Finally, in the case of asynchronous courses, having more time to evaluate the questions and formulate answers in a more constructive way was found to be beneficial.^( 25 )^

Our study had some limitations. First, we did not use objective tools to assess learning and relied only on the subjective opinions of the study participants. Secondly, the study is observational; hence, it does not prospectively present the evolution of the learning method or the changes that would occur with familiarization and adaptation to the new scenario. Additionally, we did not evaluate data considered important in the literature, such as familiarity with online classroom tools, pedagogical methods used by teachers, or whether there were changes in the methods for adapting to the online environment. Further, we did not analyze sociodemographic characteristics, which could introduce a bias within the population studied.

## CONCLUSION

Our study corroborates the existing literature that suggests remote teaching has similar evaluations to the traditional classroom system in terms of the environment, interaction, and learning from the perspectives of both students and teachers. To minimize the negative effects of remote teaching, it is important to adapt the external environment to reduce distractions, increase the interaction between students and between the teacher and students, and make classes more attractive and designed exclusively for the virtual method.
